# Pharmacokinetics of Non-Psychotropic Phytocannabinoids

**DOI:** 10.3390/pharmaceutics17020236

**Published:** 2025-02-12

**Authors:** Mariana Lacerda, Andreia Carona, Sara Castanheira, Amílcar Falcão, Joana Bicker, Ana Fortuna

**Affiliations:** 1 Laboratory of Pharmacology and Pharmaceutical Sciences, Faculty of Pharmacy, University of Coimbra, 3004-531 Coimbra, Portugal; mariana.125455@gmail.com (M.L.); afc.carona@gmail.com (A.C.); sara_castanheira@hotmail.com (S.C.); acfalcao@ff.uc.pt (A.F.); joana.bicker@gmail.com (J.B.); 2 CIBIT, Coimbra Institute for Biomedical Imaging and Translational Research, University of Coimbra, 3004-531 Coimbra, Portugal

**Keywords:** cannabidiol, cannabichromene, cannabigerol, cannabidiolic acid, cannabichromenic acid, cannabigerolic acid, pharmacokinetics

## Abstract

Cannabinoids are widely recognized for their potential therapeutic effects, making them significant and valuable candidates for medical research and applications across various fields. This review aims to analyze the pharmacokinetics of Cannabidiol (CBD), Cannabigerol (CBG), and Cannabichromene (CBC), along with their corresponding acidic forms, Cannabidiolic acid (CBDA), Cannabigerolic acid (CBGA), and Cannabichromenic acid (CBCA). Among these cannabinoids, CBD is the most extensively studied. Nevertheless, research involving all the mentioned cannabinoids has shown that their pharmacokinetic parameters are highly variable, depending significantly on factors such as dose, formulation, route of administration, and diet. Furthermore, challenges such as brain penetration and first-pass metabolism have been highlighted. In conclusion, this review demonstrates significant progress in understanding the pharmacokinetics of non-psychotropic cannabinoids. However, it also underscores the need for further research, particularly on CBG, CBC, and their respective acidic forms, with the most significant gap being in clinical investigations. Expanding these studies is essential to facilitate their optimized use in medical treatments.

## 1. Introduction

*Cannabis sativa* is a chemically complex plant from which a wide variety of compounds can be isolated. To date, 125 cannabinoids (or phytocannabinoids) have been identified and classified into eleven cannabinoid sub-classes [1]. While phytocannabinoids were once thought to be exclusively isolated from *Cannabis sativa*, currently, it is known that they also occur in flowering plants, liverworts, and even fungi [2].

The major phytocannabinoids produced by *Cannabis sativa* are Tetrahydrocannabinol (THC) and Cannabidiol (CBD) [3], with THC as the main psychotropic molecule [1]. However, minor phytocannabinoids, such as Cannabigerol (CBG), Cannabigerolic acid (CBGA), Cannabidiolic acid (CBDA), Cannabichromene (CBC), and Cannabichromenic acid (CBCA), are also expressed in lower but significant abundance [2]. In opposition to THC, these are not psychotropic drugs, making their clinical use safer compared to THC. For these reasons, the present manuscript is focused on CBD, CBG, CBC, and their respective acidic forms.

**Figure 1:**
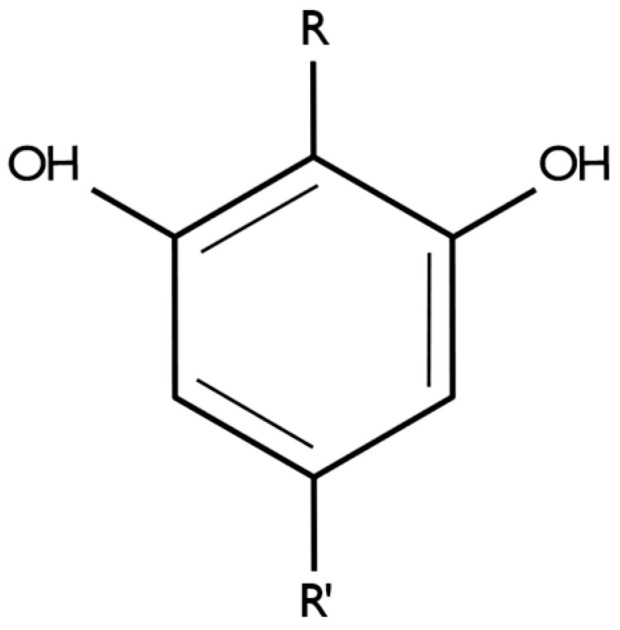
Illustration of the resorcinol core decorated with para-positioned groups. Image created using Inkscape. R—alkyl, aralkyl, or isoprenyl. R’—propyl or pentyl.

Phytocannabinoids are meroterpenoids, molecules with a resorcinyl core typically decorated with a para-positioned alkyl, aralkyl, or isoprenyl side chain [2]. In phytocannabinoids, usually one of the side chains is a propyl or pentyl [2,3] (Figure 1).

Although the core of phytocannabinoids is the same, their physicochemical properties vary because of different para-positioned groups. These properties determine the pharmacokinetics of the compounds, particularly their absorption, permeability across biological membranes, brain–blood barrier penetration, metabolism, and excretion.

According to the “Rule of Five”, intestinal absorption and drug permeability are higher for molecules that have a molecular weight lower than 500 g/mol, a Log P lower than 5, fewer than five hydrogen bond donors, fewer than 10 hydrogen bond acceptors, and a number of rotatable bonds lower than 10 [4]. However, to achieve successful brain–blood barrier penetration, molecules must also demonstrate higher lipophilicity, i.e., a Log P in the range of 1.5–2.7; lower polarity with a TPSA lower than 90 Å^2^; lower flexibility with fewer than five rotatable bonds; and a molecular weight less than 400 g/mol [5].

As summarized in Table 1, the phytocannabinoids under analysis herein present physicochemical properties that suggest that their absorption and permeability could be great, as their molecular weights are lower than 500 g/mol, and they contain fewer than 5 and 10 hydrogen bond donors and acceptors, respectively. However, their Log P is significantly above the favorable range, compromising the aqueous water solubility of the cannabinoids and, consequently, their formulation. Consequently, these phytocannabinoids are classified as class II drugs according to the Biopharmaceutics Classification System [6], presenting very low intestinal absorption. Moreover, their high lipophilicity directs the drugs quickly to the liver, where cannabinoids are extensively metabolized mainly by several enzymes of the cytochrome P450 (CYP) complex.

Although this information generally elucidates the behavior of molecules, more studies are required to accurately characterize their pharmacokinetics and biodistribution, especially because these parameters only consider passive transport. Indeed, active transport and efflux are additional important mechanisms that strongly determine drug access into the central nervous system [7].

When administered to humans, phytocannabinoids can interact with receptors in the endocannabinoid system (ECS), originally designated as such because it was discovered from studies on the mechanism of action of THC [8]. THC and its precursor, Δ9 -tetrahydrocannabivarin, bind to type 2 and type 1 cannabinoid receptors, respectively. However, the ECS is composed of more receptors, such as the G protein-coupled receptors 55 and 119, transient receptor potential vanilloid, and peroxisome proliferator-activated receptor [9]. All these receptors, endocannabinoids, enzymes responsible for their catabolism and synthesis, and genes that encode these proteins form the “endocannabinoidome” [9]. In humans, the ECS assumes multiple functions, like anti-inflammatory functions, neuroprotection, cardioprotection, and hepatoprotection [10].

Facing such a wide range of functions, cannabinoids have been largely investigated and, to date, Cannabidiol is already marked and prescribed by licensed healthcare providers. The Food and Drug Administration and European Medicines Agency approved Epidyolex^®^, composed of CBD [3,11,12], for the treatment of Lennox–Gastaut syndrome, Dravet syndrome, and seizures associated with the tuberous sclerosis complex [12]. Furthermore, in some European countries, including Portugal, Sativex^®^, which contains CBD and THC, is also approved for the treatment of spasticity, associated with multiple sclerosis [13] (Table 2).

In contrast, the interest in minor non-psychotropic phytocannabinoids is relatively recent, and, hence, most of the research is still in early stages. Table 2 summarizes the scientific evidence that supports the therapeutic potential of these cannabinoids.

Accordingly, epilepsy is one of the therapeutic indications of highest interest. Recent studies revealed that minor cannabinoids can have anticonvulsant effects. For example, an in vivo study showed that CBDA had anticonvulsant properties in rats with Dravet syndrome, reducing tonic–clonic seizures, requiring a lower dose than CBD [23]. A clinical study performed in children with Refractory Epileptic Encephalopathy evidenced a reduction in seizures after the intake of a Cannabis Herbal Extract where, beyond CBD and THC, CBC was also present and quantified [19].

In addition to epilepsy, there are other diseases in which minor cannabinoids have been demonstrated to be effective (Table 2). For example, CBC and CBG were demonstrated to be beneficial in Alzheimer’s disease [15], multiple myeloma [14], depressive disorders [15,16], and inflammatory diseases [17]. Regarding acidic cannabinoids specifically, it has been suggested that CBCA presents antibacterial activity against Methicillin-Resistant Staphylococcus aureus [18] and that CBGA and CBDA have anti-viral activity [20].

In this context, the potential of these molecules in medicine is noteworthy. However, to effectively harness the therapeutic potential of cannabinoids, a comprehensive understanding of their pharmacokinetics becomes essential. Given the challenges posed by their physicochemical properties and pharmacokinetics, such as low intestinal permeability, extensive hepatic metabolism, and short half-life time, optimizing drug formulations is required to guarantee their incorporation in the bloodstream and distribution into the brain, thus enhancing their efficacy. Furthermore, the existing pharmacokinetic data primarily focus on major cannabinoids, like THC and CBD, leaving a significant gap in the scientific knowledge regarding the pharmacokinetics of minor cannabinoids. This review discusses, for the first time, the latest results found in preclinical and clinical pharmacokinetic studies on non-psychotropic CBC, CBCA, CBD, CBDA, CBG, and CBGA to guide future research and improve therapeutic outcomes in clinical settings.

## 2. Pharmacokinetics of Cannabidiol

**Figure 2:**
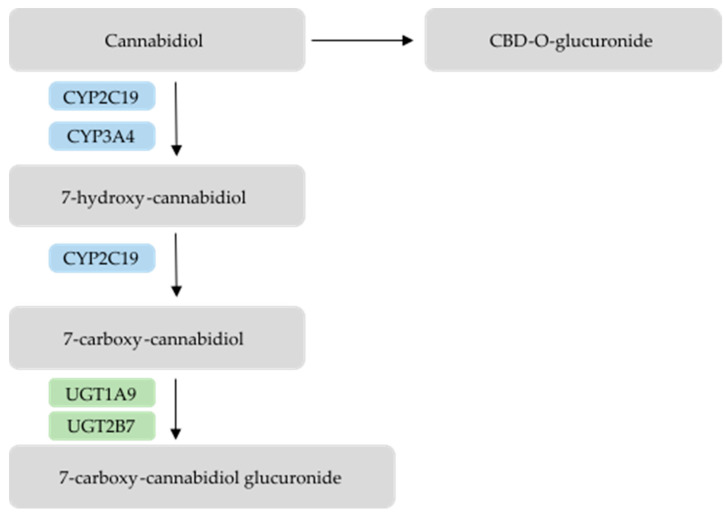
Metabolic pathways of Cannabidiol. Image adapted from Watson N. et al. [31].

Although CBD is a highly lipophilic molecule, it has very low oral bioavailability (6–19%) [25,26,27,28] not only owing to its residual dissolution in intestinal fluid, and consequently limited intestinal absorption, but also due to its extensive first-pass metabolism mediated by the isoforms CYP3A4 and CYP2C19. These enzymes catalyze the hydroxylation of CBD, forming the primary active metabolite 7-OH-CBD, which is further converted to the inactive metabolite 7-carboxy-CBD (Figure 2) [29]. Then, following glucuronidation, 7-carboxy-CBD glucuronide is formed [30]. CBD can also directly undergo glucuronidation and form CBD-O-glucuronide [29] (Figure 2). Both CBD and its metabolites are primarily excreted in feces [29]. Importantly, CYP2C19 is a polymorphic enzyme, meaning it has several alleles such as allele*1, which is associated with normal activity, allele*2, which is non-functional, and allele*17, which is hyperfunctional [31]. Depending on their inherited genotype, individuals can be normal metabolizers (genotype *1/*1), intermediate metabolizers (genotype *1/*2 or *2/*17), rapid metabolizers (genotype *1/*17), or ultrarapid metabolizers (genotype *17/*17) [32]. These genetic differences can contribute to individual variability, which may result in issues with drug efficacy and safety. This is because metabolism rates vary according to the phenotype, which in theory would lead to higher or lower concentrations of CBD in plasma. However, the relationship between CYP2C19 genetic variability and CBD metabolism is not yet fully established, as other factors may influence CYP2C19 activity and consequently the 7-hydroxylation of CBD [29,32].

Protein binding can also influence CBD efficacy, as only the unbound fraction of a drug can cross biological membranes and exert pharmacological effects. CBD is highly bound to albumin (97.4%) and to γ-globulin (84.0%) [33]. These two plasma proteins play a major role in drug transport, and their interaction with CBD regulates its distribution. The bound fraction remains confined to the intravascular space with limited access into tissues, including the brain. Indeed, under physiologic conditions, albumin does not cross the blood–brain barrier, nor the compounds bound therein. Pathologies that decrease the production of plasma proteins (e.g., hepatic cirrhosis, anemia, renal impairment) are associated with increased free drug concentrations, and consequently, side effects can occur. Also, drugs that bind more than 90% to plasma proteins can compete with CBD, reducing its binding to plasma proteins and increasing its free concentration. Hence, it is mandatory to monitor albuminemia and the pharmacotherapy of patients taking CBD.

### 2.1. Preclinical Pharmacokinetic Studies

Since CBD has low bioavailability and low water solubility, new strategies have been tested to overcome these problems, mainly including the development of specialized formulations and alternative routes of administration [28] (Table 3). Formulations, specifically nanoformulations, are one of the strategies that have led to the most relevant results. Qi Li et al. administrated nanovesicles loaded with CBD to mice and demonstrated that the plasma maximal concentrations (C_max_) of CBD obtained were almost 20 times higher than with a CBD suspension (nanovesicles C_max_ = 1943 ng/mL; suspension C_max_ = 112.0 ng/mL). On the other hand, the time to attain the C_max_ (t_max_) remained similar for both formulations after intravenous injection (t_max_ = 0.017 h) [34].

Other formulations, including oil-based, water-soluble base, and semi-solid, have also been administered orally, namely in dogs. In this line of thought, Wakshlag J. et al. tested two oil-based formulations and one chewable formulation with CBD and CBDA. The results showed no statistically significant differences between their pharmacokinetic parameters; all the formulations presented a C_max_ between 124 ng/mL and 226 ng/mL and a t_max_ between 1.5 h and 2.5 h [38]. Limsuwan S. et al. tested four formulations with distinct water solubilities, and the results demonstrated that the water-soluble base and oil-based formulations had relatively comparable pharmacokinetic plasma profiles but the nanoemulsion tended to be more quickly absorbed (nanoemulsion t_max_ = 2 h; water-soluble base formulation t_max_= 2.58 h; oil-based formulation t_max_ = 3.21 h), although to a similar extent [37]. Indeed, the C_max_ and AUC did not increase compared to the other tested formulations. As mentioned before, CBD is highly lipophilic and nanoformulations are an innovative strategy to overcome this limitation and improve CBD absorption. However, the group that received a water-soluble base formulation had the highest C_max_. Still, the mean of the results was identical to that of the oil-based formulation, so the water-soluble base formulation had a higher variation in C_max_ [37].

Regarding administration routes, intravenous administration bypasses first-pass metabolism and does not require absorption, even though it is invasive. Therefore, other administration routes have been tested and compared to the oral route [21,39,40], which is the most practical, but associated with a significant first-pass metabolism and reduced absorbed fraction.

One of the most extensively tested routes is the intranasal administration as the first-pass metabolism is reduced while brain distribution is enhanced due to direct nose-to-brain delivery pathways [21]. In fact, Pang L. et al. compared the oral and intranasal routes in mice, and the results revealed that the t_max_ of oral administration (t_max_ = 1 h) was much longer than by nasal administration (t_max_ = 0.29 h) and the C_max_ and the area under the curve (AUC) were around five and seven times lower, respectively. Therefore, the intranasal route increased the bioavailability of CBD. Regarding blood–brain barrier penetration, Pang L. et al. also demonstrated that with intranasal administration, the C_max_ attained in the brain was higher than with oral administration (intranasal C_max_ = 647.0 ng/mL; oral C_max_ = 273.0 ng/mL), supporting direct nose-to-brain CBD delivery, which overpasses the brain–blood barrier [21]. However, it would be important to compare these values with the intravenous route to confirm if CBD only reaches the brain by direct nose-to-brain connection.

Conversely, preclinical research in dogs does not corroborate this hypothesis about the intranasal or even the transmucosal route. Polidoro D. et al. compared the pharmacokinetic profiles of CBD after oral and intranasal administration, and their results demonstrated that t_max_ was faster by the intranasal route (0.49 h vs 3.5 h) but the C_max_ and AUC remained unchanged [39]. The transmucosal route was also compared to the oral route by Rocca G. et al., and the C_max_ and AUC by transmucosal administration were very similar [40]. The results of these studies do not confirm that intranasal and transmucosal administration can bypass first-pass metabolism in dogs [39,40]. In fact, it is well known that CYP enzymes are also expressed in the nasal mucosa [41], highlighting that the metabolic stability of CBD strongly compromises its bioavailability. On the other hand, the anatomy of the nasal cavity is highly different between humans, dogs, pigs, and rodents. As the nasal cavity size decreases from humans to rodents, the area of the olfactory epithelium decreases, and consequently, nose-to-brain delivery is hampered. Another relevant point is that the tested nasal formulation used a solvent mixture of 50:50 NaCl 0.9% and Polyethylene Glycol (PEG). However, it is believed that PEG-only formulations are associated with better CBD permeation. Additionally, the dogs in this study were not sedated. This lack of sedation allowed natural behaviors such as the sneezing, spillage, or swallowing of the formulation, potentially impacting the accuracy of absorption and bioavailability [39]. Regarding the transmucosal route, CBD was probably swallowed and absorbed at the gastrointestinal tract level [41]. These details may justify why the intranasal and transmucosal routes did not reveal better pharmacokinetics parameters than oral administration in dogs. Although the oral route does not prevent the first-pass metabolism, through this route, it was demonstrated that CBD is rapidly, but variably, absorbed in dogs, with a t_max_ ranging from 1.9 h to 3.5 h [36,39,40]. Chicoine A. et al. also concluded that blood concentrations of CBD increased non-linearly with the oral dose in dogs, probably owing to the saturation of CYP enzymes and plasma proteins [36]. To this day, this phenomenon has not yet been investigated for intranasal administration.

Importantly, it was demonstrated that, when administered to pregnant mice, CBD attains the fetus, namely the liver, brain, and gastrointestinal tract, within 15 min after administration [35]. This finding is particularly significant given the increasing use of CBD supplements by pregnant women, often due to its antiemetic and anxiolytic properties [35], especially in formulations with CBD that are not subjected to medical prescription. However, despite these perceived therapeutic benefits, recent in vivo and clinical studies have raised concerns. Prenatal exposure to cannabis may be associated with mood and behavioral alterations, which could be related to affective mental disorders, depressive symptoms, and ADHD [42,43]. In addition, cannabis use during pregnancy has also been associated with restricted fetal growth, low birth weight, shorter birth length, and reduced head circumference [43,44]. These findings underscore the need for caution and further investigation into the use of CBD and cannabis-related products during pregnancy.

### 2.2. Clinical Pharmacokinetic Studies

The clinical pharmacokinetic parameters of CBD are summarized in Table 4. The majority of the studies aimed to compare the pharmacokinetics of CBD when loaded in different formulations and elucidate the impact of the administered dose, diet, and renal impairment on CBD pharmacokinetics.

As emphasized before, developing the most appropriate formulation is crucial for ensuring optimal bioavailability, and this concept is present in the preclinical and clinical development phases. Even though the results obtained in dogs suggested that there were no differences between the CBD formulations, the clinical studies had distinct results. Izgelov D. et al. compared a formulation in which CBD was dissolved in sesame oil with a self-nanoemulsifying drug delivery system. No significant differences were found in the C_max_ and AUC, but both formulations led to higher C_max_ and AUC values and a shortened t_max_ compared to the powder form [45]. Sesame oil contributes to CBD lymphatic absorption [30], but self-nanoemulsifying drug delivery systems are an innovative strategy that enhances drug solubility and also contributes to lymphatic absorption [49]. On the other hand, Abbotts K. et al. tested five different CBD formulations, and their results highlighted that the four water-soluble formulations had the highest C_max_ and the shortest t_max_ compared to the oil-based formulation. Among the four water-soluble formulations under investigation, the one with the best absorption was the water-soluble formulation with maltodextrin [50]. The results from Hobbs J. et al. corroborated that a water-soluble formulation of CBD is more bioavailable than a non-water-soluble formulation of CBD. In this case, they used the same excipients, but the water-soluble formulation was emulsified and homogenized to increase water solubility [51]. All of these water-soluble formulations were designed using polymer inclusion complexes or nanoemulsions, which helped enhance CBD’s bioavailability, solubility, and stability [28]. However, the perfect formulation has not yet been achieved, though innovative strategies are being tested.

Regarding administration routes, the formulations of CBD already present in clinics are administered by the oral or oromucosal route, but first-pass metabolism and lower brain targeting are known to be a concern. Therefore, the oral route was compared to the inhalation and sublingual ones. The inhalation route can bypass first-pass metabolism, and as expected, the C_max_ of CBD was 71-fold greater after inhalation than that of oral administration while administering a 24-fold lower dose. Beyond that, the t_max_ was 30 times faster when inhaled, allowing a faster onset of action [52]. Furthermore, there was a reduction in the inactive metabolite 7-COOH-CBD, indicating that more active CBD circulated in the blood [52]. The sublingual and oral routes were compared by Hosseini A. et al. [53]. Accordingly, their formulation allowed the direct absorption of CBD into the bloodstream through the sublingual mucosa, thus preventing the first-pass metabolism [28]. Their results showed that the C_max_ of the sublingual route was nearly 30% greater and their AUC was only 10% greater compared to the oral route [53]. Notably, CBD was not the only cannabinoid present in the formulation, as it contained an extract from a *Cannabis sativa* cultivar (LINNEA 315CSE) [53]. The potential interaction between these compounds cannot be discarded, as it may have influenced the pharmacokinetics and pharmacodynamics of the cannabis-based product [36]. The literature suggests that multi-compound cannabis-based formulations, like this one, could enhance therapeutic effects through a phenomenon known as the entourage effect. However, this effect remains controversial and is widely debated within the scientific community due to the limited evidence supporting it [54].

Despite the challenges of administering CBD orally, according to Peters E. et al., there was no accumulation of CBD once they measured the pharmacokinetic parameters on day 1 and day 7 of their study, and the values did not increase throughout that period of time [48]. In their study, four different doses were administered to humans (120 mg CBD and 5.4 mg THC; 240 mg CBD and 10.8 mg THC; 360 mg CBD and 16.2 mg THC; 480 mg CBD and 21.6 mg THC) and, unlike the findings in dogs, their results demonstrated that the C_max_ of CBD was proportional to the initial dose, and the t_max_ was between 4 h and 6 h, independent of the administered dose.

Another relevant topic of investigation regards the diet’s effect on CBD pharmacokinetics. Various studies proved that a high-lipidic meal increases the C_max_ and AUC of CBD [46,49,50], probably owing to intestinal lymphatic transport. Additionally, the largest intestinal transit time and the increase in bile salts associated with the ingestion of a high-lipidic meal also contribute to enhancing the absorption of lipophilic drugs [55]. In support of this hypothesis, Crockett J. et al. demonstrated that a high-fat meal significantly increased both the C_max_ and AUC compared to other diets, such as low-fat meals and whole milk-based diets, which showed similar increases in the C_max_ and AUC. The fasted state yielded the lowest values, while alcohol intake resulted in only minimal improvements relative to fasting [49].

Several drugs are excreted by the kidneys, requiring dose adjustments in people with renal impairment. As highlighted before, the excretion of CBD occurs mainly through the liver, and the majority is excreted in feces. However, a lower percentage is excreted by the kidneys [56]. That is why Tayo B. et al. investigated if renal adjustment was needed. Their results concluded that CBD does not require these adjustments because no significant differences were observed in the C_max,_ AUC, or t_max_ between subjects with mild, moderate, and severe renal impairment [51].

## 3. Pharmacokinetics of Cannabichromene

The pharmacokinetics of CBC have not yet been thoroughly studied, with scarce preclinical and clinical studies available in the literature (Table 5).

CBC is a cannabinoid of which there is limited knowledge about its metabolism. However, there are in vitro and in vivo studies that suggest it is mainly metabolized by CYP2C9 through hydroxylation and epoxidation, resulting in two principal metabolites, 6′,7′-epoxy-CBC and 8′-hydroxy-CBC [59]. In vitro studies have also concluded that CBC undergoes glucuronidation catalyzed by UGTs 1A1, 1A8, 1A9, and 2B7 [60]. This means that CBC is susceptible to first-pass metabolism, and administration routes that do not avoid these reactions can compromise its bioavailability.

Although the oral route is subject to first-pass metabolism, it remains the most extensively studied method for administering CBC, probably because it is rapidly absorbed, with a t_max_ between 1.5 h and 3 h in rats, and does not seem to suffer accumulation at least after 14 days of administration [57].

Importantly, considering the reported therapeutic potential of CBC for central nervous system disorders such as Alzheimer’s disease and depression [15], CBC seems to successfully cross the blood–brain barrier and accumulate in the brain. Specifically, it was demonstrated in rats that on the first administration day, the concentrations ranged from 1.5 to 61.7 ng/g, increasing to 2.8 to 104.0 ng/g on the 14th day [57]. Rapid absorption through the oral route was observed not only in studies involving rats but also in studies with dogs. In mice, the t_max_ ranged between 1.5 h and 3 h, and in dogs, it ranged from 1.8 h to 2.5 h [36,57]. An important point to consider is that in mice the formulation administered had only one cannabinoid, CBC [57], but in dogs, the formulation was an herbal extract of *Cannabis sativa* [36]. In other words, this second formulation loaded more than one cannabinoid, and that might have led to interactions that would influence their pharmacokinetics [61]. However, the use of multi-cannabinoid formulations is common due to the proposed entourage effect. As previously discussed for CBD, it is believed that combining the presence of multiple cannabinoids enhances therapeutic efficacy, but this is not yet confirmed.

In clinical practice, an oral formulation with Spectrum Yellow oil loaded with CBD, THC, and CBC demonstrated that the t_max_ of CBC was similar to that observed in preclinical studies (t_max_ between 2.3 h and 3.4 h). It is important to note that once again, a formulation containing multiple cannabinoids was administered, meaning that interactions between them cannot be discarded. Nevertheless, the results of this study provide valuable insights into the pharmacokinetic profile of CBC when co-administered with other cannabinoids. Notably, the dose of CBD was 18 times higher than that of CBC, yet the AUC of CBD was only 6.6–9.8 times greater than that of CBC [48,58]. Similarly, the dose of THC was comparable to that of CBC, but THC was quantifiable in fewer plasma samples than CBC. These findings suggest that CBC achieves higher concentrations in the bloodstream, which could result from several factors: greater absorption, reduced plasma protein binding, slower metabolism, or active transport mechanisms that might be influenced by the presence of other cannabinoids [58]. Further studies are necessary to investigate these possibilities. Nonetheless, this result is particularly significant for therapeutic strategies targeting the specific effects of CBC.

## 4. Pharmacokinetics of Cannabigerol

Like CBC, the metabolism and pharmacokinetic profile of CBG are not extensively described in the literature (Table 6). 

When it comes to metabolism, in vitro studies suggest that CBG is hydroxylated by CYP2B6, CYP2C9, CYP2C19, and CYP2D6, and they also underscore glucuronidation by UGT1A9 and UGT2B7 [60]. Most of these enzymes are polymorphic, contributing to a considerable variation in the metabolic rate of CBG and, consequently, of its plasma concentration and exposure. A clinical study with *Cannabis sativa* smokers suggested that CBG excretion occurs mainly in the form of glucuronic metabolite through renal excretion [64].

Since CBG undergoes first-pass metabolism, preclinical studies have been mainly carried out comparing the pharmacokinetics of CBG after oral and intraperitoneal administrations. In mice, the absorption of CBG is rapid in both routes; however, plasma and brain concentrations are significantly higher after intraperitoneal injection compared to oral administration, with brain/plasma ratios of 0.77 and 0.15, respectively. This means that CBG access into the brain is more limited after oral administration even if normalized by plasma concentrations, comprising its therapeutic effect [7]. In rats, the absorption profile mirrors what was observed in mice: rapid absorption via both oral and intraperitoneal routes. Curiously, although intraperitoneal administration attained a higher C_max_ in plasma compared to oral administration, the concentration of CBG in the brain was only slightly higher with intraperitoneal administration than with oral administration. Interestingly, the authors detected CBG in plasma 24 h after intraperitoneal administration, whereas with oral administration, CBG levels were undetectable after 24 h [7], suggesting that when administered by oral gavage, CBG is more rapidly eliminated, probably due to first-pass metabolism.

Clinical studies further enrich the understanding of CBG pharmacokinetics; however, there is limited research in this area. Newmeyer M. et al. and Swortwood M. et al. developed studies to clarify the pharmacokinetics of some cannabinoids from an initial dose of extracts of Cannabis sativa with 50.6 mg of THC and 1.5 mg of CBD [62,63]. Therefore, their results do not correspond to isolated CBG. Accordingly, the plasma C_max_ was lower when administered by vaporization than by smoking, probably due to inefficient CBG volatilization during vaporization [62]. Although smoking is not a medical administration route, it is the most common administration route for Cannabis sativa when it comes to recreational use, justifying this study. On the other hand, Swortwood M. measured pharmacokinetic parameters through smoking, inhalation, and the oral route but in the oral fluid. Their results demonstrated that the C_max_ of CBG through vaporization was similar to oral administration and lower than when the formulation was smoked [63]. Regarding the oral route, it was expected that the concentration would be lower once CBG underwent first-pass metabolism, so the results are unsurprising. In agreement, another study by Newmeyer M. et al. demonstrated that the same dose of CBG was absent in the plasma after the oral dose [65], suggesting that oral bioavailability may be limited due to first-pass metabolism.

Despite CBG not being as well studied as CBD, Story G. studied the impact of diet on CBG pharmacokinetics, and the results were similar to those for CBD. The high-lipidic meal improved the pharmacokinetic parameters [66]. So, the diet can be a strategy to improve the bioavailability of CBG when it is administered by the oral route like it is for CBD.

## 5. Pharmacokinetics of Acidic Cannabinoids

The pharmacokinetics of acidic cannabinoids remain largely underexplored, with only a few available in vivo preclinical and clinical studies (Table 7).

CBCA, CBDA, and CBGA are the acidic forms of CBC, CBD, and CBG, respectively. Their pharmacokinetic profiles and metabolism are less studied than their neutral forms. The metabolism of these acidic forms was not found in the literature, and though it is known that the conversion from the acidic forms to their neutral forms occurs through heat, it is important to understand if it occurs in vivo. For instance, in mice, the conversion of CBCA and CBGA in their decarboxylated forms, CBC and CBG, respectively, did not occur. However, when CBDA was administered, CBD was detected in plasma at 0.5% of the CBDA concentration, suggesting that CBDA may have been converted to CBD in vivo [23]. This potential metabolic interconversion raises important considerations for therapeutic use, especially if the objective is an acidic cannabinoid-specific effect.

Only Lyndsey et al. characterized the pharmacokinetic profiles of CBCA, CBGA, and CBDA in mice after their intraperitoneal administration as a suspension dissolved in a vegetable oil vehicle [23]. Starting with CBCA, it showed rapid absorption, with a t_max_ of 30 min and a short half-life time of 24 min. Regarding brain tissue, CBCA was not measurable [23]. The pharmacokinetic profile of CBGA was slightly different, the t_max_ was a little bit longer than CBCA (t_max_= 45 min), and curiously, the peak in the brain concentration preceded the plasma peak. Despite this, both the C_max_ and AUC values in the brain were lower than those in plasma [23].

On the other hand, CBDA revealed rapid absorption, with a t_max_ of 30 min and a slower brain penetration (t_max_ of 45 min) and low exposure (given by the C_max_ and AUC) [23]. The results suggest that acidic cannabinoids administered in an oil vehicle have poor brain permeability [23], probably owing to their negatively charged carboxylic acid moiety at physiological pH, impairing passive permeation across the blood–brain barrier [5]. In addition, CBDA was administered in a Tween-based vehicle to compare the pharmacokinetic profiles. Interestingly, when CBDA was administered using the Tween-based vehicle, the brain/plasma ratio increased, suggesting better brain permeation and highlighting the influence of the vehicle on CBDA pharmacokinetics. Some authors hypothesized that nonionic surfactants disrupt the tight junctions in the blood–brain barrier; however, that cannot be the mechanism presented here, as Tetrahydrocannabinolic acid (THCA) did not attain the brain with the same vehicle [23]. Another plausible explanation suggests that CBDA is a substrate of the P-glycoprotein, and the nonionic surfactant inhibits this efflux transporter [23,67], thereby enhancing drug availability.

Although CBDA is an acidic cannabinoid, it has exhibited more extensive research coverage than the other cannabinoids, and several variables were tested, such as different animal species, administration routes, and vehicles, to understand its pharmacokinetic profile.

To elucidate the impact of formulation, Wakshlag J. et al., as seen before, compared three formulations with CBD and CBDA administered orally to dogs. The first formulation had triglyceride oil, the second lecithin and sesame oil, and the last was a chewable formulation. CBDA, like CBD, showed no significant differences in its C_max_, t_max_, and AUC values among the three formulations. However, its C_max_ and AUC were found to be twice as high as those of CBD (Table 3) [38], which can be an added value if therapeutics of CBDA are desired. Interestingly, Johns T. et al. administered two different doses (4 mg/kg and 8 mg/kg) of CBD and CBDA-rich hemp oil in *Macaca fascicularis*, and the results showed that the CBDA concentrations (4 mg/kg: C_max_ = 456.75 ng/mL; 8 mg/kg: C_max_ = 807.33 ng/mL) were higher than those of CBD (4 mg/kg: C_max_ = 15.98 ng/mL; 8 mg/kg: C_max_ = 22.31 ng/mL), corroborating the potential of CBDA as an effective cannabinoid in therapeutic applications, as it demonstrates higher bioavailability [67]. This discrepancy may be attributed to differences in absorption, distribution, metabolism, or elimination. Further in vitro and in vivo studies are required to clarify these metabolic aspects.

Busardò F. et al. conducted a clinical study investigating the disposition of CBDA and other cannabinoids in oral fluid and serum following the use of vaporized medical cannabis. Their findings confirmed that CBDA was absorbed and detectable in both oral fluid and serum. However, the concentration of CBDA was significantly lower than that of CBD, likely due to the decarboxylation of the acidic precursor during the vaporization process [68]. In mice, the conversion of CBDA to CBD was observed, raising an intriguing question: does this conversion, in this clinical study, occur exclusively due to vaporization, or does it also take place within the human body?” Therefore, it would be interesting to design clinical studies exploring alternative administration routes that do not involve vaporization or heat. Such studies should focus exclusively on CBDA to determine if its conversion to CBD occurs within the human body.

## 6. Conclusion and Future Perspectives

This review highlights significant advancements in understanding the pharmacokinetics of non-psychotropic cannabinoids, including CBC, CBG, and CBD, along with their acidic forms. Despite their therapeutic potential, the physicochemical properties of these molecules lead to considerable challenges, such as poor intestinal absorption, extensive first-pass metabolism, low bioavailability, and variable brain penetration, all of which limit their effective application in clinical settings. CBD is the most studied cannabinoid, and to improve its absorption and bioavailability, new formulations, including nanoemulsions and water-soluble systems, were tested and revealed promising results. Moreover, alternative administration routes, such as intranasal or sublingual delivery, have demonstrated great results in bypassing first-pass metabolism, yet they require deeper investigation to establish efficacy across different species and conditions.

Regarding minor cannabinoids, such as CBG, CBC, and their acidic forms, there is a substantial gap in knowledge concerning their pharmacokinetics. These cannabinoids warrant attention as they have therapeutic potential in diseases like cancer, epilepsy, and depression. Understanding their pharmacokinetics is crucial for identifying the best administration route, formulation, and dosing strategies to maximize their therapeutic effect and safety. Moreover, elucidating their plasma protein binding, metabolism, and interactions with transporters is essential as these influence bioavailability and clinical applicability.

Future investigations should aim to bridge these gaps by focusing on the pharmacokinetics of minor cannabinoids, particularly through the exploration of novel formulations and alternative administration routes. Given that minor cannabinoids also undergo first-pass metabolism, oral administration may be compromised. Intranasal and sublingual routes, which have shown promising results for CBD, could offer improved bioavailability and should be further explored for minor cannabinoids as well. Additionally, future studies should also explore how cannabinoids interact with each other, particularly in formulations containing multiple cannabinoids, as well as the influence on pharmacokinetics of variables such as diet, drug–drug interactions, age, sex, and other individual characteristics. Investigating these features will provide a more comprehensive understanding of how these substances behave in vivo, increasing their translation potential.

## Figures and Tables

**Table 1:** Physicochemical properties of Cannabichromene (CBC), Cannabidiolic acid (CBCA), Cannabidiol (CBD), Cannabidiolic acid (CBDA), Cannabigerol (CBG), and Cannabigerolic acid (CBGA).

Cannabinoid	Molecular Weight (g/mol)	Log P	Hydrogen Bond Donor	Hydrogen Bond Acceptor	TPSA (Å^2^)	Rotatable Bonds
CBC	314.5	6.9	1	2	29.5	7
CBCA	358.5	6.9	2	4	66.8	8
CBD	314.5	6.5	2	2	40.5	6
CBDA	358.5	6.6	3	4	77.8	7
CBG	316.5	7.4	2	2	40.5	9
CBGA	360.5	7.5	3	4	77.8	10

Log P—partition coefficient; TPSA—Topological Polar Surface Area.

**Table 2:** Main therapeutic potentials of each cannabinoid.

Cannabinoid	Therapeutic Potential	Reference
Cannabichromene	Alzheimer’s disease Cancer Depression Inflammation	[14,15,16,17]
Cannabichromenic acid	Bacterial infection	[18]
Cannabidiol	Alzheimer’s disease Convulsions Depressive disorders Inflammation Neuropathic pain Viral infection Post-traumatic stress disorder	[15,16,17,19,20,21,22]
Cannabidiolic acid	Convulsions Viral infection	[20,21,22,23]
Cannabigerol	Alzheimer’s disease Cancer Depressive disorders Inflammation	[14,15,16,17]
Cannabigerolic acid	Convulsions Viral infection	[20,21,22,23,24]

**Table 3:** Preclinical pharmacokinetic studies developed for Cannabidiol.

Population		AR	Matrix	Dose (mg/kg)	Time Points (h)	Pharmacokinetic Parameters			Observations	Ref
Species	Sample Size					C_max_ (ng/mL)	t_max_ (h)	AUC_t_ (ng × h/mL)		
Mice	6	Intravenous	Plasma	20	0–24	CBD suspension			This study compared the pharmacokinetics of a suspension of CBD and a nanovesicle formulation. Despite the t_max_ being the same, the C_max_ and AUC of the nanovesicle formulation were approximately 20 times and 30 times bigger, respectively.	[34]
						112.0	0.017	70.96		
						Nanovesicles				
						1943	0.017	2389		
	12	Oral	Plasma	30	0.08–12	236.2	1	428.02	With nasal administration, the C_max_ in the brain was higher, meaning it is better for achieving a brain-targeting effect.	[21]
	30	Oral	Brain		0.08–12	273.0	1	684.26		
			Liver			4631	0.88	13,723		
	12	Intranasal	Plasma		0.08–12	1205	0.29	2968.3		
	30	Intranasal	Brain		0.08–12	647.0	0.38	2138.8		
			Liver			2088	2.25	10,685		
	5	Intravenous (Tail vein of the pregnant mice)	Maternal plasma	10	0.25–12	2615.3	0	1295.2	After maternal administration, CBD rapidly reached the fetus (starting after 15 min), with a transference rate of 66.9% of the AUC. Regarding organs, the transference was also rapid, starting 15 min after administration. The transfer ratio increased during the initial 4 h period following the administration of CBD and after that declined.	[35]
	15		Fetus’s bodies		0.25–12	598.7	-	866.4		
			Fetus’s brain			-	-	1078.4		
			Fetus’s liver			-	-	1519.2		
			Fetus’s GI tract			-	-	668.8		
Dogs	13	Oral	Blood	2 CBD; 0.1 THC; 0.4 CBC	0–48.0	213	2.0	759	Oral absorption was rapid, around 2 h for all doses. C_max_ increased with dose.	[36]
				5 CBD; 0.25 THC; 1 CBC		838	1.9	2935		
				10 CBD; 0.5 THC; 2.0 CBC		1868	2.3	7239		
	7	Oral	Plasma	5 OM	0–30	270.10	3.21	1432.06	OM formulation had the biggest AUC and CM presented the lowest AUC. The kinetic profiles of the CBD in the liquid forms (OM, NM, WM) were relatively similar.	[37]
	6			5 NM		175.35	2.0	853.29		
	6			5 WM		314.30	2.58	1158.98		
	6			5 mg CM		92.29	2.83	296.05		
	6	Oral	Serum	1 CBD 1 CBDA	0–24	A 145	1.5	635	CBDA absorption was twice that of CBD absorption.	[38]
						B 124	2.0	683		
						C 226	2.5	826		
	6	Oral	Plasma	100 mg CBD	0.25–60	216.76	3.50	1376.03 ^a^	In intranasal administration, the first-pass hepatic metabolism was avoided, so it was expected that C_max_ and AUC would be greater. However, that hypothesis did not correspond to the results.	[39]
		Intranasal		20 mg CBD		27.96	0.49	61.3 ^a^		
	6	Oral	Plasma	1 CBD	0.25–10	206.77	2.17	453.17	C_max_ and AUC were similar between the oral and oral–transmucosal administrations, which suggests that CBD may have a low absorption through the oral mucosa. In the oral–transmucosal administration, CBD was probably swallowed and absorbed at the gastrointestinal tract level.	[40]
		Transmucosal				200.33	1.92	536.05		

^a^—area under the curve from time 0 to infinity; AUC_t_—AUC from time 0 to the last time point; AR—administration route; CBC—Cannabichromene; CBD—Cannabidiol; CBDA—Cannabidiolic acid; C_max_—maximal concentration; CM—semi-solid base; GI—gastrointestinal; A—oil contained 28 mg/mL of CBD, 29 mg/mL of CBDA, 1 mg/mL of THC, 0.8 mg/mL THCA, 0.7 mg/mL of CBGA, and 1.3 mg/mL CBC; B—same as Form 1 but 25% of the base oil was from sunflower lecithin; C—∼5 mg of CBDA and 5 mg of CBD; NM—nanoemulsion base; OM—oil base; Ref—references; THC—Tetrahydrocannabinol; t_max_—time to maximal concentration; WM—water-soluble.

**Table 4:** Clinical studies developed for Cannabidiol (CBD).

Sample Size	AR	Matrix	Dose (mg/kg)	Time Points (h)	Pharmacokinetic Parameters			Observations	Ref
					C_max_ (ng/mL)	t_max_ (h)	AUC_t_ (ng × h/mL)		
12 per group	Oral	Plasma	90 CBD powder	2.75–24	0.8	8.4	8	CBD in powder form had lower bioavailability than in sesame oil or SNEDDS formulations. Powder form had a delayed t_max._	[45]
			90 CBD sesame oil		14	4	66		
			90 CBD SNEDDS		18	2	61		
14	Oral	Plasma	30 CBD	0.16–4	A 0.5	1.94	1.04	Water-soluble formulation had higher C_max_ and AUC values and faster t_max._	[46]
					B 3.1	0.59	4.54		
					C 2.2	0.86	3.48		
					D 0.4	2.16	0.77		
					E 1.8	0.63	2.96		
5 per group	Oral	Plasma	30 CBD	0–6	F 2.82	0.9	408.11	Water-soluble CBD presented a C_max_ and AUC higher than lipid-soluble CBD.	[47]
					G 0.65	1.5	90.52		
8 per group	Oral	Plasma	120 mg CBD; 5.4 mg THC	1–12	Dia 1			There was proportionality in CBD exposure. There was no considerable accumulation of CBD from day 1 to day 7. Steady-state conditions were achieved within 7 days.	[48]
					8.9	4.0	34.8		
			240 mg CBD; 10.8 mg THC		7.2	4.0	29.7		
			360 mg CBD; 16.2 mg THC		10.9	6.0	51.6		
			480 mg CBD; 21.6 mg THC		23.3	5.0	117.5		
			120 mg CBD; 5.4 mg THC	1–16	Dia 7				
					9.6	4.0	62.2		
			240 mg CBD; 10.8 mg THC		18.3	4.0	110.2		
			360 mg CBD; 16.2 mg THC		23.9	4.0	175.8		
			480 mg CBD; 21.6 mg THC		36.3	5.0	263.3		
29 Fasted	Oral	Plasma	750 CBD	0.5–96	187	0.67	1077	The C_max_ and AUC of CBD were bigger with a high-lipidic meal, followed by a low lipidic meal, whole milk, and finally alcohol. Lower exposure occurs in the fasted state.	[49]
15 High					1050	0.05	4584		
14 Low					722	0.75	3202		
15 Milk					527	0.83	2450		
14 Alcohol					354	0.83	1676		
18 per group	Oral	Plasma	100 CBD	0–58	13.7	2.9	-	Dietary, oral formulation factors, and route of administration influenced CBD concentrations.	[50]
8 Mild *	Oral	Plasma	200 CBD	0.25–48	200	2.5	671	There were no notable differences in C_max_ and AUC between normal people and people with renal impairment.	[51]
8 Moderate *					172	2.0	530		
8 Severe *					155	2.5	532		
8 Healthy *					153	2.5	464		
10	Oral	Plasma	50 CBD	0.83–12	6.30	2.03	20.05	Although the dose by inhalation was lower, the C_max_ was 3 times bigger and quicker than the oral formulation.	[52]
10	Inhalation		2.1 CBD		18.78	0.063	7.66		
12	Sublingual	Plasma	25 CBD	0.25–24	9.1	4.5	31.1	Sublingual 25 mg CBD presented a CBD C_max_ twice the oromucosal values normalized to the same dose and a 25% greater AUC. Sublingual 50 mg CBD had a C_max_ nearly 30% greater and only a 10% bigger AUC than the oral formulation.	[53]
	Sublingual		50 CBD		15.0	4.1	67.3		
	Oral		50 CBD		14.0	5.2	69.8		
	Oromucosal		20 CBD ** 21.6 THC		4.6	4.5	26.6		

A—formulation was not water-soluble. Contained medium-chain triglyceride coconut oil; AR—administration route; AUC_t_—area under the curve from time 0 to the last time point; B—formulation water-soluble. Contains Arabic gum and maltodextrin; C—Water-soluble formulation. Contain Arabic gum and sorbitol; C_max_—maximal concentration; CBD—Cannabidiol; D—formulation is not water-soluble. Pure CBD as a crystalline powder; E—formulation water-soluble. Contains sorbitol; F—water-soluble CBD; G—lipid-soluble CBD; SNEDDS—self-nanoemulsified drug delivery system; Ref—references; THC—Tetrahydrocannabinol; t_max_—time to maximal concentration; * renal impairment; ** values for 20 mg CBD were normalized to 25 mg CBD.

**Table 5:** In vivo preclinical and clinical pharmacokinetic studies developed for Cannabichromene.

Population		AR	Matrix	Dose (mg/kg)		Time Points (h)	Pharmacokinetic Parameters			Observations	Ref
Species	Sample Size						C_max_ (ng/mL)	t_max_ (h)	AUC_t_ (ng × h/mL)		
Rats	36	Oral	Plasma	Day 1	3.2 CBC	0.75–24	29.72	2.25	59.56	CBC was detected in plasma at the first time point and after 24 h, after a single dose on the first day, and after 14 days of repeated doses. In both cases, the t_max_ was between 1.5 and 3 h. There was no detection of notable accumulation after day 14. CBC was also measured in the brain, and concentrations on day 14 were bigger than on day 1 for several doses, suggesting accumulation.	[57]
					10 CBC		58.35	1.5	301.42		
					17 CBC		92.22	2.25	565.83		
					22 CBC		123.12	3	762.01		
					32 CBC		207.67	2.25	1236.72		
					100 CBC		448.33	1.5	2376.14		
		Oral	Plasma	Day 14	3.2 CBC	0.75–24	27.43	1.5	102.30		
					10 CBC		80.83	3	493.79		
					17 CBC		139.90	3	845.09		
					22 CBC		126.50	1.5	673.44		
					32 CBC		199.50	1.5	1056.20		
					100 CBC		363.83	2.25	2319.68		
Beagle-cross dogs	13	Oral	Plasma	2 CBD, 0.1 THC, 0.4 CBC		0–48.0 *	17.7	2.5	53.0	The t_max_ was around 2 h for all doses, but the C_max_ differed a lot between them.	[36]
				5 CBD, 0.25 THC, 1 CBC			100.9	1.8	220.3		
				10 CBD, 0.5 THC, 2 CBC			191.6	2.3	449.4		
Human	43	Oral	Plasma	120 mg CBD, 5.4 mg THC, 6.6 mg CBC		0–12	2.4	3.2	2.2	This study concluded that CBC might possess preferential absorption over CBD and THC when administered together.	[58]
				240 mg CBD; 10.8 mg THC; 13.2 mg CBC			3.6	2.3	5.6		
				360 mg CBD; 16.2 mg THC; 19.8 mg CBC			4.8	4.3	9.0		
				480 mg CBD; 21.6 mg THC; 26.4 mg CBC			6.6	3.4	17.6		

AR—administration route; AUCt—area under the curve from time 0 to the last time point; C_max_—maximal concentration; CBC—Cannabichromene; CBD—Cannabidiol; THC—Tetrahydrocannabinol; Ref—reference; t_max_—time to maximal concentration; * at the lowest dose, samples were only measured up to 24 h.

**Table 6:** In vivo preclinical and clinical pharmacokinetic studies developed for Cannabigerol.

Population		AR	Matrix	Dose (mg/kg)	Time Points (h)	Pharmacokinetic Parameters			Observations	Ref
Species	Sample Size					C_max_ (ng/mL)	t_max_ (h)	AUC_t_ (ng × h/mL)		
Mouse	10	Oral	Plasma	120 CBG	0.5–24	670	0.5	1120 ^a^	Intraperitoneal administration resulted in significantly higher concentrations than oral administration in mice. Intraperitoneal administrations yielded bigger plasma and brain concentrations in rats than oral administrations. In intraperitoneal administration, the C_max_ in the brain was twice as high in mice than in rats, but penetration was slower.	[7]
		IP	Plasma			40,800	2	92,850 ^a^		
		Oral	Brain			420	1	≤1430 ^a^		
		IP	Brain			3480	2	18,620 ^a^		
Rat	5	Oral	Plasma			1050	0.5	1770 ^a^		
		IP	Plasma			810	1	≤10,280 ^a^		
		Oral	Brain			970	2	3410 ^a^		
		IP	Brain			1230	1	16,530 ^a^		
Human	10 FCS	Smoked	Plasma	50.6 mg THC, 1.5 mg CBD, 3.3 mg CBN	0–50	6.9	0.12	-	C_max_ was significantly greater after smoking compared to vaporization. This probably happened due to inefficient CBG volatilization during vaporization.	[62]
	5 OCS					3.0	0.10	-		
Human	10 FCS	Inhalation by vaporization	Plasma	50.6 mg THC, 1.5 mg CBD, 3.3 mg CBN		3.0	0.08	-		
	5 OCS					2.2	0.08	-		
Human	11 FCS	Smoked	Oral Fluid	50.6 mg THC 1.5 mg CBD	1.5 (prior)–50	165	0.17	-	Occasional cannabis smokers, when cannabis was vaporized, had a C_max_ value similar to oral administration, probably due to inefficient vaporization.	[63]
	9 OCS					118	0.17	-		
	11 FCS	Inhalation by vaporization	Oral Fluid			87.4	0.17	-		
	9 OCS					24.4	0.17	-		
	11 FCS	Oral	Oral Fluid			17.0	0.41	-		
	9 OCS					11.9	0.47	-		

^a^ Area Under the Curve from time 0 to infinity; AR—administration route; AUC_t_—area under the curve from time 0 to the last time point; C_max_—maximal concentration; CBD—Cannabidiol; CBG—Cannabigerol; CBN—Cannabinol; FCS—frequent cannabis smokers; OCS—occasional cannabis smokers; Ref—references; THC—Tetrahydrocannabinol; t_max_—time to maximal concentration.

**Table 7:** In vivo preclinical and clinical pharmacokinetic studies developed for acidic forms.

Population		AR	Matrix	Doses (mg/Kg)	Time Points (h)	Pharmacokinetic Parameters				Observations	Ref
Species	Sample Size					C_max_ (ng/mL)		t_max_ (h)	AUC_t_ (ng × h/mL)		
Mouse Model of Dravet syndrome	3–5 per time point	IP	Plasma	5 CBCA	0.25–2	3300		0.5	2883	CBCA intraperitoneal administration in a vegetable oil vehicle revealed t_max_ = 30 min in plasma, which means it had rapid absorption. CBCA was not detectable in the brain. That was predictable due to its carboxylic acid moiety.	[23]
		IP	Plasma	10 CBGA		63,500		0.75	99,333	Surprisingly, the t_max_ in the brain was lower than the t_max_ in plasma, so absorption occurred faster in the brain. Furthermore, the C_max_ and AUC in the brain were lower than in plasma.	
		IP	Brain			2.3		0.5	1566 (per mg of brain)		
		IP	Plasma	10 CBDA		Vegetable oil vehicle				CBDA intraperitoneal administration in an oil vehicle revealed t_max_= 30 min in plasma, which means it had rapid absorption. CBDA was detected in the brain and t_max_ was 45 min, slower than in plasma. Absorption of CBDA increased in the brain when administered in a Tween-based vehicle instead of an oil vehicle.	
						29,600		0.5	78,316		
						Tween-based vehicle					
						17,600		0.25	9966		
		IP	Brain			Vegetable oil vehicle					
						2 (per mg of brain)		0.75	3.05 (per mg of brain)		
						Tween-based vehicle					
						13.2(per mg of brain)		0.5	19.03 (per mg of brain)		
Beagle dog	6	Oral	Serum	1 CBD	0–24	A	383	1	1.018	CBDA absorption was twice that of CBD absorption. The C_max_ was possibly missed because it was at the first time point. The formulation with lecithin and a sesame oil base had the biggest AUC.	[38]
				1 CBDA		B	386	1.2	1.610		
						C	510	2.3	1.407		
*Macaca fascicularis*	8	Oral	Plasma	4 CBD/CBDA	0–24	456.75		0.5	838.28	C_max_ and AUC were greater at 8 mg/kg than at a 4 mg/kg dose. C_max_ is less than doubled at 2 doses, and AUC is more than doubled so it does not have linearity. The results present considerable inter-subject variability.	[67]
				8 CBD/CBDA		807.33		1	2759.26		
Human	15	Inhalation by vaporization	Serum	8 mg THC + THCA 6 mg CBD + CBDA	0.25 (prior)–24	34.54		0.17	63.80	CBDA presented 3-times bigger C_max_ and 4-times lower t_max_ than CBD in oral fluid. In serum, CBDA C_max_ was one-third of CBD C_max,_ and t_max_ was equal for both.	[68]
		Inhalation by vaporization	Oral Fuid			10.22		0.17	11.36		

AUC_t_—area under the curve from time 0 to the last time point; AR—administration route; C_max_—maximal concentration; CBCA—Cannabichromenic acid; CBD—Cannabidiol; CBDA—Cannabidiolic acid; CBGA—Cannabigerolic acid; M—matrix; Ref—references; THC—Tetrahydrocannabinol; THCA—Tetrahydrocannabinolic acid; t_max_—time to maximal concentration; A—oil contained 28 mg/mL of CBD, 29 mg/mL of CBDA, 1 mg/mL of THC, 0.8 mg/mL THCA, 0.7 mg/mL of CBGA, and 1.3 mg/mL CBC; B—was the same as Form 1 regarding cannabinoid concentration except that 25% of the base oil was from sunflower lecithin; C—∼5 mg of CBDA and 5 mg of CBD.

## Data Availability

No new data were created.

## References

[1] Radwan M.M., Chandra S., Gul S., Elsohly M.A. (2021). Cannabinoids, Phenolics, Terpenes and Alkaloids of Cannabis. Molecules.

[2] Gülck T., Møller B.L. (2020). Phytocannabinoids: Origins and Biosynthesis. Trends Plant Sci..

[3] Walsh K.B., McKinney A.E., Holmes A.E. (2021). Minor Cannabinoids: Biosynthesis, Molecular Pharmacology and Potential Therapeutic Uses. Front. Pharmacol..

[4] Lipinski C.A., Dominy B.W., Feeney P.J. (1997). Drug Delivery Reviews Experimental and Computational Approaches to Estimate Solubility and Permeability in Drug Discovery and Development Settings. Adv. Drug Deliv. Rev..

[5] Pajouhesh H., Lenz G.R. (2005). Medicinal Chemical Properties of Successful Central Nervous System Drugs. NeuroRX.

[6] Reddy T.S., Zomer R., Mantri N. (2023). Nanoformulations as a Strategy to Overcome the Delivery Limitations of Cannabinoids. Phytother. Res..

[7] Deiana S., Watanabe A., Yamasaki Y., Amada N., Arthur M., Fleming S., Woodcock H., Dorward P., Pigliacampo B., Close S. (2012). Plasma and Brain Pharmacokinetic Profile of Cannabidiol (CBD), Cannabidivarine (CBDV), Δ9-Tetrahydrocannabivarin (THCV) and Cannabigerol (CBG) in Rats and Mice Following Oral and Intraperitoneal Administration and CBD Action on Obsessive-Compulsive Behaviour. Psychopharmacology.

[8] Di Marzo V., Piscitelli F. (2015). The Endocannabinoid System and Its Modulation by Phytocannabinoids. Neurotherapeutics.

[9] Di Salvo A., Conti M.B., della Rocca G. (2023). Pharmacokinetics, Efficacy, and Safety of Cannabidiol in Dogs: An Update of Current Knowledge. Front. Vet. Sci..

[10] Matei D., Trofin D., Iordan D.A., Onu I., Condurache I., Ionite C., Buculei I. (2023). The Endocannabinoid System and Physical Exercise. Int. J. Mol. Sci..

[11] Food and Drug Administration FDA Regulation of Cannabis and Cannabis-Derived Products, Including Cannabidiol. https://www.fda.gov/news-events/public-health-focus/fda-regulation-cannabis-and-cannabis-derived-products-including-cannabidiol-cbd.

[12] (2019). Infarmed Relatório Público De Avaliação Do Pedido De Comparticipação De Medicamento Para Uso Humano. https://www.infarmed.pt/documents/15786/1437513/Relat%FF%FFrio%2Bp%FF%FFblico%2Bde%2Bavalia%FF%FF%FF%FFo%2Bdo%2Bmedicamento%2BSativex%2B2019/c055642c-92fe-4e84-9da6-f72f3a9c0e06.

[13] Aguzzi C., Zeppa L., Morelli M.B., Marinelli O., Giangrossi M., Amantini C., Santoni G., Sazzad H., Nabissi M. (2024). Anticancer Effect of Minor Phytocannabinoids in Preclinical Models of Multiple Myeloma. BioFactors.

[14] Fonseca C., Ettcheto M., Bicker J., Fernandes M.J., Falcão A., Camins A., Fortuna A. (2023). Under the Umbrella of Depression and Alzheimer’s Disease Physiopathology: Can Cannabinoids Be a Dual-Pleiotropic Therapy?. Ageing Res. Rev..

[15] El-Alfy A.T., Ivey K., Robinson K., Ahmed S., Radwan M., Slade D., Khan I., ElSohly M., Ross S. (2010). Antidepressant-like Effect of Δ9-Tetrahydrocannabinol and Other Cannabinoids Isolated from *Cannabis sativa* L.. Pharmacol. Biochem. Behav..

[16] Blevins L.K., Bach A.P., Crawford R.B., Zhou J., Henriquez J.E., Rizzo M.D., Sermet S., Khan D.M.I.O., Turner H., Small-Howard A.L. (2022). Evaluation of the Anti-Inflammatory Effects of Selected Cannabinoids and Terpenes from Cannabis Sativa Employing Human Primary Leukocytes. Food Chem. Toxicol..

[17] Galletta M., Reekie T.A., Nagalingam G., Bottomley A.L., Harry E.J., Kassiou M., Triccas J.A. (2020). Rapid Antibacterial Activity of Cannabichromenic Acid against Methicillin-Resistant *Staphylococcus aureus*. Antibiotics.

[18] Huntsman R.J., Tang-Wai R., Alcorn J., Vuong S., Acton B., Corley S., Laprairie R., Lyon A.W., Meier S., Mousseau D.D. (2019). Dosage Related Efficacy and Tolerability of Cannabidiol in Children with Treatment-Resistant Epileptic Encephalopathy: Preliminary Results of the CARE-E Study. Front. Neurol..

[19] van Breemen R.B., Simchuk D. (2023). Antiviral Activities of Hemp Cannabinoids. Clin. Sci..

[20] Pang L., Zhu S., Ma J., Zhu L., Liu Y., Ou G., Li R., Wang Y., Liang Y., Jin X. (2021). Intranasal Temperature-Sensitive Hydrogels of Cannabidiol Inclusion Complex for the Treatment of Post-Traumatic Stress Disorder. Acta Pharm. Sin. B.

[21] Hansen J.S., Boix F., Hasselstrøm J.B., Sørensen L.K., Kjolby M., Gustavsen S., Hansen R.M., Petersen T., Sellebjerg F., Kasch H. (2024). Pharmacokinetics and Pharmacodynamics of Cannabis-based Medicine in a Patient Population Included in a Randomized, Placebo-controlled, Clinical Trial. Clin. Transl. Sci..

[22] Anderson L.L., Low I.K., Banister S.D., McGregor I.S., Arnold J.C. (2019). Pharmacokinetics of Phytocannabinoid Acids and Anticonvulsant Effect of Cannabidiolic Acid in a Mouse Model of Dravet Syndrome. J. Nat. Prod..

[23] Anderson L.L., Heblinski M., Absalom N.L., Hawkins N.A., Bowen M.T., Benson M.J., Zhang F., Bahceci D., Doohan P.T., Chebib M. (2021). Cannabigerolic Acid, a Major Biosynthetic Precursor Molecule in Cannabis, Exhibits Divergent Effects on Seizures in Mouse Models of Epilepsy. Br. J. Pharmacol..

[24] Perucca E., Bialer M. (2020). Critical Aspects Affecting Cannabidiol Oral Bioavailability and Metabolic Elimination, and Related Clinical Implications. CNS Drugs.

[25] Millar S.A., Stone N.L., Yates A.S., O’Sullivan S.E. (2018). A Systematic Review on the Pharmacokinetics of Cannabidiol in Humans. Front. Pharmacol..

[26] Millar S.A., Maguire R.F., Yates A.S., O’Sullivan S.E. (2020). Towards Better Delivery of Cannabidiol (CBD). Pharmaceuticals.

[27] Hossain K.R., Alghalayini A., Valenzuela S.M. (2023). Current Challenges and Opportunities for Improved Cannabidiol Solubility. Int. J. Mol. Sci..

[28] Beers J.L., Fu D., Jackson K.D. (2021). Cytochrome P450–Catalyzed Metabolism of Cannabidiol to the Active Metabolite 7-Hydroxy-Cannabidiol. Drug Metab. Dispos..

[29] Nasrin S., Watson C.J.W., Perez-Paramo Y.X., Lazarus P. (2021). Cannabinoid Metabolites as Inhibitors of Major Hepatic CYP450 Enzymes, with Implications for Cannabis-Drug Interactions. Drug Metab. Dispos..

[30] Moriyama B., Obeng A.O., Barbarino J., Penzak S.R., Henning S.A., Scott S.A., Agúndez J.A.G., Wingard J.R., McLeod H.L., Klein T.E. (2017). Clinical Pharmacogenetics Implementation Consortium (CPIC) Guidelines for CYP2C19 and Voriconazole Therapy. Clin. Pharmacol. Ther..

[31] Batinic A., Sutlović D., Kuret S., Matana A., Kumric M., Bozic J., Dujic Z. (2023). Trial of a Novel Oral Cannabinoid Formulation in Patients with Hypertension: A Double-Blind, Placebo-Controlled Pharmacogenetic Study. Pharmaceuticals.

[32] Liu C., Cai A., Li H., Deng N., Cho B.P., Seeram N.P., Ma H. (2022). Characterization of Molecular Interactions between Cannabidiol and Human Plasma Proteins (Serum Albumin and γ-Globulin) by Surface Plasmon Resonance, Microcalorimetry, and Molecular Docking. J. Pharm. Biomed. Anal..

[33] Li Q., Wang C., Hu J., Jiao W., Tang Z., Song X., Wu Y., Dai J., Gao P., Du L. (2023). Cannabidiol–Loaded Biomimetic Macrophage Membrane Vesicles against Post–Traumatic Stress Disorder Assisted by Ultrasound. Int. J. Pharm..

[34] Ochiai W., Kitaoka S., Kawamura T., Hatogai J., Harada S., Iizuka M., Ariumi M., Takano S., Nagai T., Sasatsu M. (2021). Maternal and Fetal Pharmacokinetic Analysis of Cannabidiol during Pregnancy in Mice. Drug Metab. Dispos..

[35] Chicoine A., Illing K., Vuong S., Pinto K.R., Alcorn J., Cosford K. (2020). Pharmacokinetic and Safety Evaluation of Various Oral Doses of a Novel 1:20 THC:CBD Cannabis Herbal Extract in Dogs. Front. Vet. Sci..

[36] Limsuwan S., Phonsatta N., Panya A., Asasutjarit R., Tansakul N. (2024). Pharmacokinetics Behavior of Four Cannabidiol Preparations Following Single Oral Administration in Dogs. Front. Vet. Sci..

[37] Wakshlag J.J., Schwark W.S., Deabold K.A., Talsma B.N., Cital S., Lyubimov A., Iqbal A., Zakharov A. (2020). Pharmacokinetics of Cannabidiol, Cannabidiolic Acid, Δ9-Tetrahydrocannabinol, Tetrahydrocannabinolic Acid and Related Metabolites in Canine Serum After Dosing with Three Oral Forms of Hemp Extract. Front. Vet. Sci..

[38] Polidoro D., Temmerman R., Devreese M., Charalambous M., Van Ham L., Cornelis I., Broeckx B.J.G., Mandigers P.J.J., Fischer A., Storch J. (2022). Pharmacokinetics of Cannabidiol Following Intranasal, Intrarectal, and Oral Administration in Healthy Dogs. Front. Vet. Sci..

[39] della Rocca G., Paoletti F., Conti M.B., Galarini R., Chiaradia E., Sforna M., Dall’Aglio C., Polisca A., Di Salvo A. (2023). Pharmacokinetics of Cannabidiol Following Single Oral and Oral Transmucosal Administration in Dogs. Front. Vet. Sci..

[40] Oliveira P., Fortuna A., Alves G., Falcao A. (2016). Drug-Metabolizing Enzymes and Efflux Transporters in Nasal Epithelium: Influence on the Bioavailability of Intranasally Administered Drugs. Curr. Drug Metab..

[41] Huizink A.C., Mulder E.J.H. (2006). Maternal Smoking, Drinking or Cannabis Use during Pregnancy and Neurobehavioral and Cognitive Functioning in Human Offspring. Neurosci. Biobehav. Rev..

[42] Roncero C., Valriberas-Herrero I., Mezzatesta-Gava M., Villegas J.L., Aguilar L., Grau-López L. (2020). Cannabis Use during Pregnancy and Its Relationship with Fetal Developmental Outcomes and Psychiatric Disorders. A Systematic Review. Reprod. Health.

[43] El Marroun H., Tiemeier H., Steegers E.A.P., Jaddoe V.W.V., Hofman A., Verhulst F.C., van den Brink W., Huizink A.C. (2009). Intrauterine Cannabis Exposure Affects Fetal Growth Trajectories: The Generation R Study. J. Am. Acad. Child Adolesc. Psychiatry.

[44] Izgelov D., Davidson E., Barasch D., Regev A., Domb A.J., Hoffman A. (2020). Pharmacokinetic Investigation of Synthetic Cannabidiol Oral Formulations in Healthy Volunteers. Eur. J. Pharm. Biopharm..

[45] Abbotts K.S.S., Ewell T.R., Butterklee H.M., Bomar M.C., Akagi N., Dooley G.P., Bell C. (2022). Cannabidiol and Cannabidiol Metabolites: Pharmacokinetics, Interaction with Food, and Influence on Liver Function. Nutrients.

[46] Hobbs J.M., Vazquez A.R., Remijan N.D., Trotter R.E., McMillan T.V., Freedman K.E., Wei Y., Woelfel K.A., Arnold O.R., Wolfe L.M. (2020). Evaluation of Pharmacokinetics and Acute Anti-inflammatory Potential of Two Oral Cannabidiol Preparations in Healthy Adults. Phytother. Res..

[47] Peters E.N., Mosesova I., MacNair L., Vandrey R., Land M.H., Ware M.A., Turcotte C., Bonn-Miller M.O. (2022). Safety, Pharmacokinetics and Pharmacodynamics of Spectrum Yellow Oil in Healthy Participants. J. Anal. Toxicol..

[48] Crockett J., Critchley D., Tayo B., Berwaerts J., Morrison G. (2020). A Phase 1, Randomized, Pharmacokinetic Trial of the Effect of Different Meal Compositions, Whole Milk, and Alcohol on Cannabidiol Exposure and Safety in Healthy Subjects. Epilepsia.

[49] Bergeria C.L., Spindle T.R., Cone E.J., Sholler D., Goffi E., Mitchell J.M., Winecker R.E., Bigelow G.E., Flegel R., Vandrey R. (2022). Pharmacokinetic Profile of ∆9-Tetrahydrocannabinol, Cannabidiol and Metabolites in Blood Following Vaporization and Oral Ingestion of Cannabidiol Products. J. Anal. Toxicol..

[50] Tayo B., Taylor L., Sahebkar F., Morrison G. (2020). A Phase I, Open-Label, Parallel-Group, Single-Dose Trial of the Pharmacokinetics, Safety, and Tolerability of Cannabidiol in Subjects with Mild to Severe Renal Impairment. Clin. Pharmacokinet..

[51] Devinsky O., Kraft K., Rusch L., Fein M., Leone-Bay A. (2021). Improved Bioavailability with Dry Powder Cannabidiol Inhalation: A Phase 1 Clinical Study. J. Pharm. Sci..

[52] Hosseini A., McLachlan A.J., Lickliter J.D. (2021). A Phase I Trial of the Safety, Tolerability and Pharmacokinetics of Cannabidiol Administered as Single-dose Oil Solution and Single and Multiple Doses of a Sublingual Wafer in Healthy Volunteers. Br. J. Clin. Pharmacol..

[53] Christensen C., Rose M., Cornett C., Allesø M. (2023). Decoding the Postulated Entourage Effect of Medicinal Cannabis: What It Is and What It Isn’t. Biomedicines.

[54] Zgair A., Wong J.C., Lee J.B., Mistry J., Sivak O., Wasan K.M., Hennig I.M., Barrett D.A., Constantinescu C.S., Fischer P.M. (2016). Dietary Fats and Pharmaceutical Lipid Excipients Increase Systemic Exposure to Orally Administered Cannabis and Cannabis-Based Medicines. Am. J. Transl. Res..

[55] Martinez Naya N., Kelly J., Corna G., Golino M., Polizio A.H., Abbate A., Toldo S., Mezzaroma E. (2024). An Overview of Cannabidiol as a Multifunctional Drug: Pharmacokinetics and Cellular Effects. Molecules.

[56] Moore C.F., Weerts E.M., Kulpa J., Schwotzer D., Dye W., Jantzi J., McDonald J.D., Lefever T.W., Bonn-Miller M.O. (2023). Pharmacokinetics of Oral Minor Cannabinoids in Blood and Brain. Cannabis Cannabinoid Res..

[57] Peters E.N., MacNair L., Mosesova I., Christians U., Sempio C., Klawitter J., Land M.H., Ware M.A., Turcotte C., Bonn-Miller M.O. (2022). Pharmacokinetics of Cannabichromene in a Medical Cannabis Product Also Containing Cannabidiol and Δ9-Tetrahydrocannabinol: A Pilot Study. Eur. J. Clin. Pharmacol..

[58] Roy P., Maturano J., Hasdemir H., Lopez A., Xu F., Hellman J., Tajkhorshid E., Sarlah D., Das A. (2024). Elucidating the Mechanism of Metabolism of Cannabichromene by Human Cytochrome P450s. J. Nat. Prod..

[59] Havlasek J., Vrba J., Zatloukalova M., Papouskova B., Modriansky M., Storch J., Vacek J. (2023). Hepatic Biotransformation of Non-Psychotropic Phytocannabinoids and Activity Screening on Cytochromes P450 and UDP-Glucuronosyltransferases. Toxicol. Appl. Pharmacol..

[60] Pavlovic R., Nenna G., Calvi L., Panseri S., Borgonovo G., Giupponi L., Cannazza G., Giorgi A. (2018). Quality Traits of “Cannabidiol Oils”: Cannabinoids Content, Terpene Fingerprint and Oxidation Stability of European Commercially Available Preparations. Molecules.

[61] Newmeyer M.N., Swortwood M.J., Barnes A.J., Abulseoud O.A., Scheidweiler K.B., Huestis M.A. (2016). Free and Glucuronide Whole Blood Cannabinoids’ Pharmacokinetics after Controlled Smoked, Vaporized, and Oral Cannabis Administration in Frequent and Occasional Cannabis Users: Identification of Recent Cannabis Intake. Clin. Chem..

[62] Swortwood M.J., Newmeyer M.N., Andersson M., Abulseoud O.A., Scheidweiler K.B., Huestis M.A. (2017). Cannabinoid Disposition in Oral Fluid after Controlled Smoked, Vaporized, and Oral Cannabis Administration. Drug Test. Anal..

[63] Hidvégi E., Somogyi G.P. (2010). Detection of Cannabigerol and Its Presumptive Metabolite in Human Urine After Cannabis Consumption. Pharmazie.

[64] Newmeyer M.N., Swortwood M.J., Andersson M., Abulseoud O.A., Scheidweiler K.B., Huestis M.A. (2017). Cannabis Edibles: Blood and Oral Fluid Cannabinoid Pharmacokinetics and Evaluation of Oral Fluid Screening Devices for Predicting Δ9-Tetrahydrocannabinol in Blood and Oral Fluid Following Cannabis Brownie Administration. Clin. Chem..

[65] Story G., Lee J., Cohen G., Rani A., Doherty J., Sela D.A. (2024). Impact of Dietary Fat and Oral Delivery System on Cannabigerol Pharmacokinetics in Adults. Cannabis Cannabinoid Res..

[66] Johns T.N., Wakshlag J.J., Lyubimov A.V., Zakharov A., Burnside W.M. (2023). Pharmacokinetics of Cannabidiol-/Cannabidiolic Acid-Rich Hemp Oil in Juvenile Cynomolgus Macaques (*Macaca fascicularis*). Front. Vet. Sci..

[67] Busardò F.P., Pérez-Acevedo A.P., Pacifici R., Mannocchi G., Gottardi M., Papaseit E., Pérez-Mañá C., Martin S., Poyatos L., Pichini S. (2021). Disposition of Phytocannabinoids, Their Acidic Precursors and Their Metabolites in Biological Matrices of Healthy Individuals Treated with Vaporized Medical Cannabis. Pharmaceuticals.

[68] ten Tije A.J., Verweij J., Loos W.J., Sparreboom A. (2003). Pharmacological Effects of Formulation Vehicles. Clin. Pharmacokinet..

